# A Review of Privacy Enhancement Methods for Federated Learning in Healthcare Systems

**DOI:** 10.3390/ijerph20156539

**Published:** 2023-08-07

**Authors:** Xin Gu, Fariza Sabrina, Zongwen Fan, Shaleeza Sohail

**Affiliations:** 1 School of Information Technology, King’s Own Institute, Sydney, NSW 2000, Australia; xin.gu@koi.edu.au; 2 School of Engineering and Technology, Central Queensland University, Sydney, NSW 2000, Australia; f.sabrina@cqu.edu.au; 3 College of Computer Science and Technology, Huaqiao University, Xiamen 361021, China; 4 College of Engineering, Science and Environment, The University of Newcastle, Callaghan, NSW 2308, Australia; shaleeza.sohail@newcastle.edu.au

**Keywords:** federated learning, privacy enhancement, differential privacy, homomorphic encryption, blockchain, P2PS, edge device, edge federated learning

## Abstract

Federated learning (FL) provides a distributed machine learning system that enables participants to train using local data to create a shared model by eliminating the requirement of data sharing. In healthcare systems, FL allows Medical Internet of Things (MIoT) devices and electronic health records (EHRs) to be trained locally without sending patients data to the central server. This allows healthcare decisions and diagnoses based on datasets from all participants, as well as streamlining other healthcare processes. In terms of user data privacy, this technology allows collaborative training without the need of sharing the local data with the central server. However, there are privacy challenges in FL arising from the fact that the model updates are shared between the client and the server which can be used for re-generating the client’s data, breaching privacy requirements of applications in domains like healthcare. In this paper, we have conducted a review of the literature to analyse the existing privacy and security enhancement methods proposed for FL in healthcare systems. It has been identified that the research in the domain focuses on seven techniques: Differential Privacy, Homomorphic Encryption, Blockchain, Hierarchical Approaches, Peer to Peer Sharing, Intelligence on the Edge Device, and Mixed, Hybrid and Miscellaneous Approaches. The strengths, limitations, and trade-offs of each technique were discussed, and the possible future for these seven privacy enhancement techniques for healthcare FL systems was identified.

## 1. Introduction

Federated Learning (FL) has gained immense popularity recently in academic research as well as real-world implementation. Compared to the traditional machine learning system which collects data from all sources and trains data at a central server, FL provides a distributed machine learning system that enables participants to train their local model on local data and eliminates the requirement of data sharing [1]. The participants only share the parameters of the trained model with the FL server. The FL server aggregates the model weights and then sends the new model weights back to the participants. The iterated training allows each participant to build one global model collaboratively without ever sharing the data with other participants [2].

Due to the inherent characteristics of distributed training and learning model architecture, FL has gained significant popularity in the healthcare domain. In the context of healthcare, FL allows multiple healthcare institutions to collaborate and train machine learning models based on decentralized data without the need of sharing sensitive patient information. It has been increasingly adopted and implemented in hospitals and healthcare settings. Not only serving as the academic pioneering pathway, but FL is also now being applied in real-world healthcare scenarios.

Google and Stanford Medicine conducted a study, in which FL was employed to develop a predictive model for hospital readmissions. Patient data from multiple hospitals were stored locally. Without sharing sensitive information, the distributed machine learning model trained the data stored locally from multiple hospitals. The resulting model provided personalized readmission risk assessments while ensuring patient privacy.

Clinical decision support systems adopted FL. Kyung Hee University developed a deep learning-based clinical decision support system under an FL paradigm enabled large-scale clinical data mining [3]. The objective was to assist healthcare professionals in making accurate diagnoses and treatment decisions.

FL has demonstrated potential in accelerating drug discovery and development processes. Very recently, 10 pharmaceutical companies, academic research labs, large industrial companies and startups constructed a large industry-scale FL model for drug discovery without sharing the confidential data sets [4]. Diverse patient data can be trained on the FL models to potentially achieve the identification of potential drug targets, prediction of drug efficacy, and optimization of treatment protocols, while confidentiality of the patient data are preserved.

FL has been leveraged for collaborative disease diagnosis and detection, such as diabetic retinopathy from fundus images [5]. Several hospitals shared their locally trained models for diagnosing diabetic retinopathy. The models were aggregated through FL to achieve an accurate and generalized model for detecting the disease from fundus images.

In population health management, FL has been investigated, such as predicting clinical outcomes in patients with COVID-19 [6]. This application helps in proactive intervention, disease prevention, and resource allocation while ensuring privacy and compliance with data protection regulations.

## 2. Existing Reviews

Driven by the recent advances in FL in the healthcare domain, quite a few reviews and surveys have been done in the area of FL in healthcare applications in the last three years. In this section, we provide a brief overview of these recent reviews and surveys.

Four review studies discussed and evaluated FL from the aspects of privacy and security [1,7], data heterogeneity [1], traceability and accountability [1], system architecture [1], statistical challenges [7], system challenges [7], data-centric perspectives [8], and applicability for confidential healthcare datasets [9]. In the review from Rieke et al. [1], summaries included the key factors of FL and how FL may provide a solution for future digital health. Most importantly, the review highlighted the challenges and considerations in FL systems: data heterogeneity, privacy and security, traceability and accountability, and system architecture. Xu et al. [7] carried out a survey on FL technologies within biomedical space. In their discussion, privacy-preserving issues covered secure multi-party computation and differential privacy. Shyu et al. [8] reviewed 24 papers on FL in healthcare applications, and evaluated challenges from a data perspective, including data partitioning characteristics, data distributions, data protection mechanisms, and benchmark datasets. Pfitzner et al. [9] provided a review of 80 relevant papers on FL and its applicability to confidential healthcare datasets. In this research, decentralized learning using a Blockchain or Peer-2-Peer was excluded.

Three review studies on FL are related to Electronic Health Records (EHR). Kumar and Singla [10] investigated the FL healthcare models in the fields of EHR systems, drug discovery and disease prediction systems. In this study, different FL algorithms were assessed and compared using parameters such as accuracy, precision, recall and F-score. Nguyen et al. [11] conducted a survey on the use of FL in smart healthcare. The survey was based on 16 papers. This survey also provided a review of the FL-based applications in healthcare domains including EHR data management, remote health monitoring, medical imaging, and COVID-19 detection. Antunes et al. [12] presented a systematic literature review through 44 papers on FL-based healthcare applications in the context of EHR data. The review further discussed the general architecture to facilitate the use of the FL with ML-enhanced applications.

There are a few review studies focused on their own area, such as oncology-based applications [13], IoMT-related FL [14], and security threats [15]. Chowdhury et al. [13] discussed the FL applications and algorithms in the oncology space through a systematic review of 63 papers. Ali et al. [14] conducted a survey on FL in smart IoMT healthcare systems from the perspective of privacy. The study explained the security and privacy of IoMT, and FL in IoMT. The paper also discussed FL architectures, which included privacy-enabled FL, incentive-enabled FL for IoMT and FL-enabled digital twin for IoMT. Mothukuri et al. [15] focused on privacy-specific threats associated with FL compared to security threats. In the discussion, security vulnerabilities and threats in FL are identified and examined, and privacy threats and their mitigation techniques in FL are identified and evaluated.

The key topics and the highlights of these review studies are listed in Table 1.

The existing work reviewed many different aspects of FL in healthcare systems including data, system, statistical challenges, applicability, and privacy and security.

In light of the inherent characteristics of FL architecture, a certain level of privacy is provided in shielding healthcare data from leakage by not sharing the raw data. However, the collective intelligence builder, FL, does not provide waterproof privacy protection. Specially, healthcare data including digital health records generally contain personal data that are highly private, sensitive, and confidential. The nature of healthcare data poses significant privacy risks.

Recently several studies have brought to attention that the model parameters shared by the participants within the FL systems could possibly reveal patients’ private information [17,18]. Additionally, there is no formal proof of privacy guarantees [19] for the FL process as there is a possibility of security attack due to inherent vulnerabilities associated with a distributed system. Hence, FL systems require additional mechanisms to improve privacy and security vulnerabilities when specifically used in the healthcare domain. In this paper, the focus of privacy enhancement is on privacy leakage from the model gradients in the FL systems (with some exceptions) [20].

There is no review conducted on privacy enhancement methods used in FL in healthcare systems. Therefore, our review aims to explore privacy enhancement methods used in FL in healthcare systems. Specifically, our work aims to answer the following research questions:

- RQ. 1: What are the existing privacy enhancement methods in FL in healthcare systems to make data more secure in the healthcare systems?
- RQ. 2: What are the strengths, weaknesses, and trade-offs of the existing privacy enhancement methods in FL in healthcare systems?
- RQ. 3: What is the future of privacy enhancement methods in FL in healthcare systems?

In this paper, the recent privacy enhancement approaches proposed for FL in the field of healthcare were reviewed. Only literature during the period of 2020–2023 that specifically provides options to secure FL systems by providing privacy guarantees to highly sensitive medical data was included in this review.

In Section 3, the review methodological approach is documented in detail. The results of the review, and corresponding answers to the research questions, are provided in Section 4. Section 5 summarises the findings, and provides a discussion of opportunities for privacy enhancement methods in FL in healthcare systems, and future work.

## 3. Methodology

To achieve the objectives of our review, the relevant academic publishing was collected from Scopus which has been supported as having the widest coverage in peer-reviewed scholarly journals and publications among bibliographic databases [21,22,23]. The bibliography data source and parameters of the search are listed in Table 2.

There are three key components in the search query to serve the objectives of this review. Firstly, all published work needs to be related to FL. Secondly, those published studies need to be in the health domain. Thirdly, the published studies need to be related to security, privacy or trust in the FL. The search components are listed in Table 3.

Based on the search keywords identified in the three components, the Boolean search string is constructed: TITLE-ABS-KEY (“federated learning” AND (“ehealth” OR “health”) AND (“security” OR “privacy” OR “trust”) )

Through Scopus search, 216 records were returned. The returned papers were screened, mapped, and extracted. Eventually, the assessment work of a full context review on the relevancy of these returned papers were conducted. To determine whether a paper is relevant, the paper needs to match all three search components: FL, health, and privacy/secure/trust. As a result, a set of 39 articles were identified as relevant.

During the process of review, one article in the area of intelligence on the edge device was found relevant through the reference list of an identified article. Therefore, it was included in this review. As a result, 40 relevant articles were identified and included in this review.

## 4. Results

In this section, the results of the review based on the 40 relevant articles are presented.

### 4.1. Research Question 1

The first research question is “What are the existing privacy enhancement methods in FL in healthcare systems?”

To answer this research question, the selected papers can be categorised into the following major areas based on the principle approach used for providing security and privacy to the FL system:

- Differential Privacy
- Homomorphic Encryption
- Blockchain
- Hierarchical Approaches
- Peer-to-Peer Sharing
- Intelligence on the Edge Devices
- Mixed, Hybrid and Miscellaneous Approaches

The following subsections start with a brief introduction to the basic approach and then provide the analysis and discussion on the research contribution of the selected paper in the area of privacy enhancement for FL.

#### 4.1.1. Differential Privacy

Differential privacy (DP) provides a way to securely share aggregate information from a dataset without compromising the privacy of an individual data subject. AI techniques generally require large datasets to train the models which arises privacy concerns for sensitive information maintained in the datasets. DP provides a framework to develop these intelligent algorithms with the ability to analyse privacy costs while maintaining acceptable accuracy [24]. When it comes to FL, to prevent information leakage the concept of DPis utilized by adding noise to the parameters that are exchanged by the participants before model aggregation at the server side [25]. FL provides mitigation of privacy risks as compared to centralized machine learning models but is still vulnerable to attacks when it comes to extracting information from the gradients sent by the participants to the aggregation server. DP provides means to quantify personal information disclosure when machine learning models are using personal user data. By adding a level of uncertainty to the model, privacy is provided to safeguard user information. Hence, a tradeoff between model accuracy and desired privacy level needs to be considered [26].

FL is vulnerable to privacy attacks as global model aggregation is performed at the centralized server. In the healthcare field, patient data can reveal private information and as the central server has access to the model parameters so a compromised server can be a great security threat. Therefore, an edge aggregator is proposed that introduces artificial noise to the client model parameters in three steps [27]. The introduction of this aggregator still provided good model accuracy while achieving privacy due to the introduction of the noise. This work falls under the category mentioned in Section 4.1.6 as well.

Due to the high privacy requirements associated with patients’ datasets, a security challenge is presented when feeding data into machine learning models. Statistical feature selection and differential privacy approaches are adopted [28] to reduce the data transmission and to add noise to the local models before sending those to the FL server for aggregation. The results showed good accuracy with the desired level of privacy applied to the model parameters.

Additional privacy is crucial when FL is used for COVID-19 detection using chest X-rays and symptoms information across multiple hospitals to safeguard data from malicious attackers. In addition to the measures to improve model accuracy for data, non-IID privacy preservation is achieved by adding the differential privacy stochastic gradient descent (DP-SGD) which provides required resilience to adaptive attacks auxiliary information [26]. The participants of the FL system perform perturbation on their gradients after training the model on their local data using DP-SGD. By adding random Gaussian noise to local gradients privacy preservation was achieved at the cost of model accuracy, this tradeoff is analysed. The results showed that by increasing the number of participants in the FL system high differential guarantee can be achieved with minimal effect on model accuracy [26].

The network traffic exchanged by the Internet of Health Things (IoHT) may be open to attacks that can result in privacy breaches of patients’ personal information in health systems. An FL-based anomaly detection system is proposed that uses DP to provide privacy guarantees to data owners and uses machine learning to secure network traffic [29]. A blockchain-enabled collaborative FL architecture is proposed that uses DP for safeguarding user data and blockchain for secure sharing of data [30]. The theoretical analysis showed that the system provides security to the processes that require sharing of data.

DP provides the required privacy guarantees at the cost of reducing the prediction accuracy of the machine learning model. An end-to-end pipeline consisting of FL and DR is proposed for health data streams using a clustering mechanism to reduce model training time with high accuracy [31]. DP mechanism is used by the system to provide privacy guarantees and results showed that prediction accuracy only decreases by 2% for the trained model due to this change. Similarly, DP is used to provide privacy guarantees when FL is used in a distributed environment to train multiple instance learning models to classify medical images [32]. The results showed that the distributed federated training with privacy guarantees achieved comparable performance to the conventional centralized training approach.

In a smart home environment where multiple IoT devices are used to collect personal health and other data, privacy preservation is very crucial. Alzheimer’s disease detector is designed that uses FL and considers user audio collected by different IoT devices to detect the disease. FL system uses a novel DP-based asynchronous privacy-preserving aggregation framework to provide guaranteed privacy. A secret sharing technique is proposed which protects the confidentiality of the weighted average at every round in the FL system and is based on the discrete logarithm difficulty hypothesis. The results show good performance of the disease detection module with high-security protection.

#### 4.1.2. Homomorphic Encryption

Homomorphic encryption is a special type of encryption where ciphertext can be used to apply mathematical operations without requiring deciphering of the text [33]. Full harmonic encryption can be very suitable for encrypting the model updates from the participant to the server in FL systems which can be aggregated without requiring decyphering.

Homomorphic encryption is used to provide privacy while training predictive models on diabetes data using the FL system. A third-party collaborator is introduced to generate public and secret key pairs and to send the public key to the participants [34]. Participants use this key to perform homomorphic encryption on model updates before sending those to the server. FL server can aggregate the updates without deciphering those and send back the updated weights. The proposed federated forest algorithm showed good performance in terms of privacy protection and prediction accuracy. Similarly, a cancer text classification system based on FL is proposed that uses homomorphic encryption to secure model parameter exchange between FL participants and server [35].

A multi-modal machine learning model is proposed to effectively solve the problem of data islands and uses homomorphic encryption to securely share data required for training machine learning models [36]. A vertical FL system is developed that compresses and decompresses the feature value while transmitting those to improve the safety of the feature value in the transmission process. The results showed the feasibility of the proposed multimodal learning and provided better accuracy than unimodal options. A secure FL system is proposed to protect against data island-level poisoning attacks for medical diagnosis based on multikey computation [37]. For keeping the FL system secure a trimmed optimization method is used to provide protection against a range of data island-level poisoning attacks in the FL and for privacy preservation pocket diagnosis is proposed based on multi keys using homomorphic encryption. A privacy-aware and resource-saving collaborative learning protocol is proposed to provide privacy for EHRs when the FL system is engaged in resource-saving scenario [38]. The resource savings at the participants are provided by outsourcing the major learning component of the neural network model to the cloud servers. Lightweight data perturbation and packed partially homomorphic encryption are used to protect the privacy of data during transmissions and also the privacy of the model updates transferred between participants and servers.

#### 4.1.3. Blockchain

FL allows multiple participants to train a shared model without the need to exchange their raw data. One of the significant privacy challenges FL is facing is to ensure that the participating devices are trustworthy and not malicious. Corrupted data from a malicious intentional participant can have a major impact on the model’s outcome. Additionally, there is the issue of fairness and transparency in the distribution of rewards for participants. Blockchain technology can help overcome these challenges to manage the FL process without the need for intermediaries. Blockchain technology provides a decentralized ledger that can store transactions securely and transparently. In the context of FL, blockchain technology can be used to create a transparent and tamper-proof record of the data provided by each participant [11,39].

In an FL-chain (FL with blockchain technology) system, the participating devices can be identified and verified using their public key [40]. This ensures that only trustworthy devices are allowed to participate in the learning process. Additionally, the devices can be incentivized to participate in the learning process by rewarding them with tokens that can be exchanged for goods or services.

Blockchain technology can also be used to create a transparent and decentralized model selection process and machine learning process [41]. In a traditional FL system, the model selection process is typically managed by a centralized server. However, in an FL-chain system, the selection process can be decentralized, ensuring that the decision-making process is transparent and fair. In a traditional machine learning process, the model owners have to trust the centralized platform to manage the transaction process. However, with an FL-chain, the transaction process can be managed in a decentralized and transparent way, ensuring that the transaction process is fair and trustworthy. This process can be set to fully decentralized, partially decentralized or hybrid mode.

Chang, Fang, and Sun [42] proposed an FL-chain system with an adaptive differential privacy algorithm. Technically, the system adjusts the amount of noise by adding on the gradient to achieve the balance between privacy and accuracy in a MIoT environment. The key strength of this FL-chain system is the gradient verification-based consensus protocol which is implemented to prevent malicious attacks and single point of failure.

Passerat-Palmbach et al. [43] proposed an FL-chain framework with six elements for privacy-preserving in electronic health data to avoid bias for higher security and persistence. The six elements are discoverable data and analytic process, fabricated value, compute guarantees, privacy guarantees, and data quality.

Salim and Park [44] presented a secure EHR scheme in a hospital that adopted FL and blockchain technology. In the scheme, Inter Planetary File System (IPFS) stores private data. In the IPFS, all EHR hashed addresses are recorded using a Consortium Blockchain-based network. Access to the EHR is granted by individual patients enabled by smart contracts. EHR data are trained both locally and globally.

Lakhan et al. [45] implemented fraud detection using the rule-based blockchain in the IoMT FL network. In this study, an FL-chain-enabled task scheduling (FL-BETS) framework with different dynamic heuristics was proposed. The goal of FL-BETS is to identify and ensure the privacy preservation and fraud of data at various levels.

#### 4.1.4. Hierarchical Approaches

Although FL may overcome privacy issues faced by a centralized cloud server-based data analysis approach to some extent, the existing traditional FL approach is still restrictive when data aggregation is done on a single server. Hence few works concentrated on the development of a hierarchical FL approach that permits aggregation at varying levels, facilitating collaboration among multiple parties [46,47,48].

Gupta et al. [46] proposed a hierarchical FL approach for the Internet of Medical Things (IoMT) application which allows data aggression at different levels enabling multiple healthcare organisations to collaborate with each other securely. A novel approach of grouping is proposed where the data is grouped based on different disease groups. The authors also developed a novel technique, called Federated Time Distributed (FEDTIMEDIS) Long Short-Term Memory (LSTM), for training the anomaly detection model.

Singh et al. [47] introduced a Hierarchical FL (HFL) model based on Dew-Cloud technology, which offers an enhanced level of data confidentiality for Internet of Medical Things (IoMT) applications. In this study, for detecting intrusion in incoming traffic a hierarchical long-term memory (HLSTM) model has also been deployed at distributed Dew servers. The authors claim that the proposed model can gain users’ trust in the IoMT ecosystem.

Abdellatif et al. [48] presented a hierarchical FL application that aims to enhance privacy in healthcare applications. The proposed architecture comprises different layers such as End User (EU) layer, Edge node layer, and Centralized Server layer. In this approach, EUs collaborate to train a deep learning model without sharing raw data, by connecting to the edge nodes within their communication range. These edge nodes are then connected to a centralized server. The authors highlight that their proposed method achieves efficiency by considering the varying data distributions among EUs, as well as the conditions of the wireless environment.

#### 4.1.5. Peer to Peer Sharing

Efficiency is also very important in FL. To achieve high efficiency, peer-to-peer sharing (P2PS) in FL is utilised to facilitate the exchange of model updates between participating devices and enhance the efficiency of the learning process by reducing the computational burden on centralized servers. In addition, P2PS can also improve user privacy without the need for a central intermediary to store and process sensitive data.

Gandhi et al. [35] proposed an FL system to maintain the integrity of sensitive medical data by using a few deep-learning models for cancer text classification. Their results showed that the proposed framework is effective in addressing the privacy issues of sensitive medical data for cancer text classification.

With the increasing EHRs collected from smartphones and wearable devices, Chen et al. [49] proposed an efficient and privacy-preserving system (PFL-IU), which is compatible with irrelevant updates. The results showed that the PFL-IU has good performance in terms of privacy, accuracy, convergence and efficiency.

#### 4.1.6. Intelligence on the Edge Device

In the healthcare field, edge intelligent computing is often deemed highly appropriate for use with FL as it minimizes the need to transmit patients’ private data to the cloud [50]. Border gateways collect the initial model from the cloud and then train the model with the data collected from edge intelligent devices. A mask and digital signature are used to secure model parameters after training at the gateways. The aggregator at the cloud adds a common mask vector before sending the updated model to the gateways. As no noise is added to the model parameters so the model shows higher accuracy than systems that use differential privacy for safeguarding patients’ information.

Intelligence in the edge could be improved by incorporating a data analytics framework on the edge which assists the FL module to re-train local ML models with user-customised insights [51]. Hakak et al. [51] proposed a framework that provides privacy of data by incorporating cloud, edge and application modules. By providing aggregation at the local device and central cloud level, the privacy of data is maintained. Based on application requirements local controller saves the data and the updated encrypted local weights from the local devices are aggregated locally by the local aggregator. These values are sent to the cloud aggregator for the global model update. Homomorphic encryption is used as it allows calculations to be computed over the encrypted data without the need for decryption.

Wang et al. [52] proposed a novel protocol for parameter aggression in FL and its application for edge devices that have limited capacity. The goal is to improve security while reducing communication and computational overhead. The authors has proposed a novel approach of orthogonal gradient aggregation (OGA) that can protect previously learned knowledge when the related training samples are removed and hence ensure privacy of data.

Wang et al. [50] proposes a Privacy Protection Scheme for FL under Edge Computing (PPFLEC) that uses a newly proposed privacy protection protocol based on a shared secret and weight mask. The proposed protocol uses a random mask scheme of secret sharing and focuses on achieving lightweight features to make it suitable for the Internet of Medical Things on the edge. This work also uses a hash function and a digital signature to ensure the integrity of the message, as well as protect the system from replay attacks.

In edge computing, as most computations are done at the edge using long encryption keys, this causes a large overhead for resource-constrained IoT devices. Hence, some intelligence on the edge could be a possible solution for enhancing data privacy and achieving low complexity. To achieve this goal, Akter et al. [27] proposed an edge aggregator that introduces noise to the clients’ model parameters and by employing DP while considering the privacy budget, the results showed high model accuracy.

#### 4.1.7. Mixed, Hybrid and Miscellaneous Approaches

By combining different types of privacy-preserving approaches, a hybrid solution can overcome the weaknesses of individual approaches by providing stronger techniques. For example, by combining blockchain with homomorphic encryption a decentralised, transparent privacy-preserving solution is presented for smart parking with minimum computation and communication overhead [2]. In this section, we discuss approaches that:

- have employed multiple approaches for providing privacy to FL systems
- are different from other categories and due to being the only approach of its type cannot be placed in a separate category

Instead of the commonly used differential privacy approach, the syntactic approach is proposed for FL systems that provide required guarantees for privacy and still have good model accuracy [53]. The proposed syntactic anonymity approach provides interpretable universal privacy guarantees. The training is done according to the requirements of the syntactic privacy model. The FL system used healthcare data to predict adverse drug reactions and mortality rates and benchmarked against the centralized model.

A privacy-preserving FL system that resolves the issue of irrelevant updates is proposed [49]. The system provides secure aggregation of the model updates at the FL server with a non-interactive key generation algorithm. The sign of the model parameter which is considered as the gradient’s direction determines the direction in which the model is updated and the relevance of every local update is determined by this. The results showed that the proposed system can accelerate model convergence while providing high prediction accuracy in a secure manner.

The purpose of the hybrid approaches is to improve the overall performance of the models used in FL by leveraging the strengths of different models. It can not only improve the efficiency of FL models by accelerating the convergence speed but also enhance their accuracy by improving the data quality. In addition, domain-specific knowledge can be incorporated into the training to improve the model’s robustness.

Considering the patients’ privacy, [54] applied the FL to overcome the limitations of privacy and data-sharing laws and regulations in radiology. To maintain user data privacy, an anomaly detection system was proposed in [55]. Their results showed that the proposed post-quantized FL model is able to effectively identify malicious events with high accuracy and also ensures privacy. To address the privacy-preserving analysis with horizontally partitioned data. In addition, [56] proposed creating multiple models from different families. Otoum et al. [57] proposed a solution to secure medical devices by employing transfer learning and neural networks in a federated system. The proposed model showed better performance than other models under comparison in centralized learning schemes. Chamikara et al. [58] proposed privacy-preserving method for horizontal FL systems showed comparatively better performance in terms of accuracy, efficiency, scalability, and attack resistance. Nguyen et al. [59] proposed a knowledge distillation-based decentralized FL model to ensure data privacy and protection. Even with poor-quality data, the proposed model has shown high performance. Luo et al. [60] proposed a novel method to fit the generalized linear mixed model with advantages including lossless, privacy-preserving and fast-converging for hospital profiling.

Ma et al. [61] proposed an improved federated tensor factorization to reduce the uplink communication cost. They also proved that the convergence speed of the proposed model did compromise using aggressive communication compression. Wu et al. [62] proposed the FL model to reduce communication costs by considering different techniques and results showed a huge reduction of communication cost with competitive results. To reduce the parameters shared in the FL process, Paragliola [63] proposed a method that balances model accuracy while considering costs associated with communication among participants and servers.

Han et al. [64] introduced a secure approach that preserves the accuracy of the medical data and the results verified its robustness to conventional and geometric attacks. Gong et al. [65] proposed a cloud based FL model for user behaviour sensing where LSTM is introduced to deal with the non-IID (Independent and Identically Distributed) problem of the distributed training data with its performance better than the comparing models. To address the unfairness problem in FL, Siniosoglou et al. [66] proposed an unsupervised fairness method to identify defective models with promising results. Considering the traditional decentralized FL models are sensitive to Byzantine attacks, Gouissem et al. [67] proposed a decentralized server-less FL model using dual-way update. The results showed the proposed model is effective for error propagation control and in a collaborative isolation decision of the malicious users with high efficiency.

### 4.2. Research Question 2

The second research question is “What are the strengths, weaknesses, and trade-offs of each type of the existing privacy enhancement methods in FL in healthcare systems?”

The second research question is answered based on the seven method types as summarized in the literature review in RQ1.

The key strengths of each privacy enhancement method type are listed in Table 4.

The key weaknesses of each privacy enhancement method type are listed in Table 5.

#### 4.2.1. Differential Privacy

DP provides privacy of user data as a mathematical guarantee, that from the model parameters, the individual data points cannot be distinguished in a decentralised manner as no central entity is required to manage this privacy enhancement. DP is not primarily used for securing systems but it certainly improves the security of an FL system by making it harder for the attackers to extract information from the model parameters. The noise is added to these parameters before the aggregation process which makes model inversion attacks difficult to carry out. The principles of DP are based on the quantifiable framework of privacy guarantees with a defined privacy budget and noise variance to provide trust among participants. Hence, some sharing of these initial parameters may be required but the actual processing is done by individual clients, hence, this makes it a semi-decentralised approach, which fits exactly the architecture of the FL system. DP has no contribution to data governance in an FL system, aspects like data quality, data access policies and consent management are not part of the DP approach. FL systems are based on principles of collaboration which are promoted by the use of DP in FL as it provides additional privacy for individual data sources.

Differential privacy in large-scale systems may introduce scalability issues as the amount of noise required for providing the guarantee increases with the increase in client numbers. This may impact the performance of ML algorithms in FL systems. Similarly, as the clients need to perform additional tasks for adding noise to the model parameters before sending those to the server so all DP approaches add computational overhead and increase design complexity. For resource constraint IoT devices, DP based approaches also increase energy consumption due to added tasks. As already discussed, governance is not part of any DP-based approach so that needs to be handled by adding relevant governance frameworks, policies and mechanisms for ensuring proper use of data and resources.

In FL systems the data from multiple participants are collected and shared, DP ensures the identity of any individual in the dataset cannot be reconstructed and hence provides privacy protection to user information. DP does that by introducing noise to the data, which adversely affects the prediction accuracy of the ML algorithms. Higher privacy guarantee results in higher degradation of the accuracy of the system and hence, this tradeoff needs to be considered carefully for FL systems. An optimal balance between privacy and accuracy is a critical area of future work in differential privacy and requires context awareness for applications in different domains. In the healthcare field, the accuracy of the prediction model is a critical factor when it comes to disease detection and other related applications. The healthcare FL system that provides a high privacy guarantee, and high accuracy is still a crucial requirement, hence the use of DP is very challenging and requires additional methods to mitigate the impact on system accuracy.

#### 4.2.2. Homomorphic Encryption

Homomorphic encryption matches the FL architecture in terms of decentralization as the encryption is performed at the clients, with some form of key management required. Homomorphic encryption promotes the privacy and security of FL systems as data and model parameters remain confidential throughout the learning, transfer and aggregation process. All clients may use the same encryption/decryption methods which provides transparency of the homomorphic encryption mechanism. Collaboration is supported in HE-based systems as clients keep control of their data and allow computations on the encrypted shared model information without raising privacy concerns. However, governance is something that is only partially supported as other mechanisms are required to perform tasks like key sharing, data lifecycle management and consent management etc.

Homomorphic encryption-based approaches for FL have numerous weaknesses, especially in terms of resource usage. The encryption process that clients need to perform adds design complexity, increased computational overhead and also results in increased energy consumption for resource constraint IoT devices. In FL systems with a large number of clients, scalability can be an issue as the computational requirements increase significantly due to the additional complexity of computations required on encrypted data. Privacy provided by this method also has issues like the vulnerability related to Metadata, which can still leak information and may reveal sensitive details of the data and computations. Governance is an important factor in this case as the management of encryption keys is a challenge.

For FL applications where data is distributed and the computational capacity of the participants is not known, new algorithms are required that consider optimised use of computational resources at the participants. In this context, we need to consider the type of computations required by different ML algorithms and a suite of Homomorphic algorithms may be required to optimise computational efficiency for specific machine learning models, As discussed in the previous section, scalability and privacy-accuracy trade-off needs to be considered when FL specific Homomorphic algorithms are designed.

#### 4.2.3. Blockchain

The strengths of using blockchain technology in FL systems lie in its ability to enhance privacy, security, and transparency. The inherent architecture of blockchain including the local data storage on individual devices, in addition to the cryptographic algorithms and immutability feature in the blockchain technology, provides enhanced data privacy and security. The transparent nature of the blockchain enables all participants in the FL systems to have visibility into the updates and changes made to the model. The decentralized and trust-enhancing properties of blockchain technology facilitate collaboration in FL systems. Participants have higher trust in the shared model and the integrity of the updates due to the consensus mechanism and immutability. Smart contracts automation is the embedded strength of blockchain technology. Smart contracts can be utilized to define and enforce data usage policies, ensuring that data is shared and utilized in accordance with predefined rules and regulations.

The use of blockchain technology in an FL system can have several limitations. Although blockchain technology offers enhanced privacy through decentralization and cryptographic techniques, it does not provide complete privacy protection. The transparent nature of blockchain can expose certain information. This may be harmful to sensitive data in FL-chain systems. All of the four FL-chain systems [42,43,44,45] from this review adopted additional privacy enhancement methods to improve privacy protection. Besides the integration of the additional privacy enhancement methods, the consensus process in the FL-chain systems [42,43,44,45] requires significant computational resources. When multiple participants are involved in an FL-chain, the computational overhead of maintaining the blockchain would increase dramatically. This could result in a slower training speed, impact the efficiency of the system, and face scalability challenges. Additionally, it requires a significant amount of energy to complete the computation. Blockchain networks often struggle with scalability limitations. This could be a significant drawback when a large number of participants proceed with the updates in the FL-chain systems. The consensus mechanisms can result in slower processing time and the scalability can be reduced. Incorporating blockchain into an FL system requires detailed design and integration. Developing smart contracts, defining consensus mechanisms, and ensuring compatibility with the existing FL protocols need to be included in the design and implementation. The complexity of the FL chain could increase development time and budget. From a governance perspective, to integrate blockchain technology in FL systems, reaching consensus on system updates, protocol changes, or policy modifications may require extensive coordination and agreement among participants, potentially slowing down decision-making processes.

Using blockchain technology in FL healthcare systems involves several trade-offs that need to be carefully assessed. Blockchain’s decentralized architecture enhances security and transparency. However, it comes at the cost of scalability. The transparency offered by blockchain makes all transactions visible to all participants in the network, which enhances trust and auditability but also conflicts with privacy requirements in FL systems. Cryptographic algorithms and consensus mechanisms enhance the security feature, however, it comes at a cost in terms of computational overhead. The computational complexity can impact the efficiency and speed of the FL systems, especially during the training phase. Integrating blockchain into an FL system requires significant development work and expertise. It requires smart contracts and consensus mechanisms, and interoperability with existing FL protocols. The complexity increases the challenges of long-term maintenance of the system. Time and cost are the two objectives for governance and decision-making for implementing or maintaining a system. Consensus on system updates, policy modifications, and protocol changes may require extensive coordination and agreement among participants, which may slow down the decision-making process. The trade-offs between scalability and decentralization, transparency and privacy, security and efficiency, difficulty level of implementation and integration, and governance and control need to be carefully considered when designing an FL-chain healthcare system.

#### 4.2.4. Hierarchical Approaches

Hierarchical approaches have inherent strengths in terms of decentralization and transparency as adding multiple edge servers and having a transparent grouping mechanism can make these approaches suitable for a large number of FL applications. The distributed nature of the system and collaboration at the tier level adds a privacy and security layer to the system. Governance and collaboration can be considered strengths of hierarchical systems as they provide a structural framework for managing and controlling data and grouping clients.

Scalability can be considered a strength of these approaches as with the addition of more clients, adding more edge servers can provide clear distribution of load and management overhead. However, as the number of participating clients increases computational overhead and energy consumption at the cloud and edge servers increase but will not affect the clients. However, governance and design complexity are considerable challenges in these systems as management of a large number of clients and servers and designing appropriate aggregation mechanisms are non-trivial in those cases.

For hierarchical approaches in FL, existing work focused mainly on grouping clients in different levels or providing a hierarchical architecture to ensure even distribution of the network load among all connected devices. A more detailed insight into the trade-offs of these hierarchical approaches is required in order to evaluate the security compromises related to the sensitive participant information and security threats from adversaries to different levels of the network. The introduction of multiple edge servers introduces more vulnerable entities in the system where all participant information and data need to be secured. Also, due to the introduction of multiple tiers, the global model may take longer to converge and this trade-off needs to be considered for systems requiring real-time task management.

#### 4.2.5. Peer to Peer Sharing

The P2PS technology has been widely used in FL systems, which shows multiple advantages. First of all, is the enhanced privacy. The participants can share the model updates directly with each other. The exposure of sensitive user data to third-party entities and reduces the risk of data breaches is minimised. Additionally, P2PS reduces the impact of individual failures which makes the FL systems resilient. If any participant in the FL system is unavailable, the remaining participants can continue to collaborate and contribute to the learning process. P2PS techniques utilize the computing resources across multiple participants where each participant contributes its own processing power, which can significantly improve the overall computational capabilities of the FL systems. With more participants joining the P2PS, the available computing resources increase in the FL system. Thus, the FL system has the capacity to handle large datasets or complex models. P2PS in FL allows for direct communication between participants, bypassing the need for a central server. By doing so, the P2PS is able to alleviate network congestion and reduce the overall communication latency in the system. In other words, P2PS could create a bigger and faster FL system. The last but not the least advantage of the P2PS is that they can span across different geographical locations, which allows for diverse participation in the FL system. Thus A model from a broader range of data distributions can be generated.

Although P2PS has shown many strengths in enhancing the privacy of FL systems, it is not completely free from limitations. Although the P2PS is able to provide offer scalability as mentioned in the advantages, managing large-scale FL systems with a massive number of participants can be difficult. It is very challenging to coordinate and synchronize model updates across numerous participants. In addition, the overhead of maintaining network connections increases and communication reliability decreases with the growing number of participants. The lack of centralized control or authority makes it hard to control the reliability of all participants. Direct communication among participants in P2PS architecture could bring security risks in the FL systems, such as an increased risk of data leakage and unauthorized access. In addition, some malicious participants might try to exploit vulnerabilities in the system to gain unauthorized access to sensitive user data or manipulate the learning process. Due to the decentralized mechanisms for resource discovery and P2P connections, it is difficult to discover available and suitable participants with the desired computational resources and it is challenging to identify capable and trustworthy participants in the learning process. As a result, the overall performance of the FL systems could be affected. In P2P FL systems, The communication between participants and model updates could lead to overhead and high communication costs. The transmission of large model parameters among participants results in high network bandwidth and increase communication latency.

Considering the strengths and limitations, there are some trade-offs in designing a P2P FL system. Although the P2PS is able to provide the utilization of distributed computing resources among participants, the heterogeneity of participants’ capabilities could result in imbalances of resource utilization. The FL emphasizes data privacy by keeping user data on local devices. However, P2P allows participants directly share model updates, which results in trust concerns. It is important to establish trust among participants to ensure the integrity and confidentiality of the shared data. With the lack of centralized control, the P2PS could introduce security risks. To reduce such risks, it is important to ensure the security measures, such as encryption, authentication, and so on. P2PS provides scalability by distributing computational tasks among participants, it is difficult to coordinate and synchronize the learning process as the number of participants increases. A trade-off exists between network efficiency and communication overhead in P2PS. Transferring large model parameters across participants can consume significant network bandwidth and increase latency. Balancing network efficiency and communication overhead is crucial to optimize the learning process. Careful considerations are required when addressing the trade-offs to balance resource utilization, privacy, security, scalability, and network efficiency.

#### 4.2.6. Intelligence on the Edge Device

The first and most important strength of adopting intelligence on edge devices in FL systems lies in privacy enhancement. Its decentralized architecture ensures preserving data confidentiality and compliance with regulations. Additionally, due to the distributed architecture, scalability can be achieved, allowing for larger datasets and improved model performance. Via this technique, transmitting only model updates and aggregated results reduces communication costs, saves network resources and minimizes latency. Immediate responses are enabled by the real-time processing and inference on the edge devices. System robustness and fault tolerance are improved as the edge devices can operate autonomously. Energy efficiency is improved as local processing minimizes the need for continuous network communication. These strengths make intelligence on the edge devices an optimal choice for FL systems where privacy, reduced communication, real-time processing, robustness, energy efficiency, and scalability are critical factors.

Intelligence on edge devices in FL systems also has certain limitations. The model complexity and training capabilities are restricted due to the limited computational resources provided by the edge devices. Heterogeneity among devices caused by variations in hardware and software can complicate optimization and compatibility. Model performance could be affected by the data imbalance among the distributed datasets. Network bandwidth and latency could hassle data synchronization and model updates. Security could set the alarm due to the vulnerability of edge devices, requiring robust security measures which governance is not provided by edge devices.

Certain trade-offs should be considered when designing intelligence on edge devices in FL systems. The model complexity may be limited by the device’s computational capabilities. The limited computational resources on edge devices could compromise model accuracy. As data distribution and quality may vary among devices, privacy preservation can affect model performance, potentially impacting model generalization. Energy efficiency is achieved by local processing, but complex models can consume more energy. Scalability could be affected due to heterogeneity among various types of edge devices, which requires additional efforts for compatibility. Balancing model accuracy, privacy, scalability, real-time processing, centralized control, and energy efficiency is crucial to effectively leverage intelligence on edge devices in FL systems.

#### 4.2.7. Mixed, Hybrid and Miscellaneous Approaches

Mixed, hybrid, and miscellaneous approaches try to leverage their strengths and compensate for their weaknesses so as to provide a more comprehensive and robust privacy framework. These approaches allow for fine-grained privacy control over different aspects of the FL process. They explore techniques that can provide strong privacy guarantees while still maintaining acceptable levels of model accuracy and performance. They also provide flexibility and adaptability to accommodate diverse privacy requirements and system constraints. In addition, they improve the resilience of the FL system against various privacy attacks by integrating multiple techniques. These approaches strive for compatibility and interoperability with existing FL frameworks and infrastructures and encourage continuous improvement and innovation in privacy protection. It’s worth noting that the specific strengths and effectiveness of mixed, hybrid, and miscellaneous approaches depend on the selection and combination of techniques, as well as their proper implementation and configurations. Considering the specific privacy requirements, data characteristics, and system constraints are key factors in determining the most suitable approach for a given FL system.

However, integrating multiple privacy techniques in a mixed or hybrid approach can increase the complexity of the FL system. In addition, combining different privacy techniques may introduce compatibility and interoperability challenges. Privacy techniques such as encryption, secure multiparty computation, or differential privacy can introduce computational complexity and communication requirements. The combination of privacy techniques may introduce a trade-off between privacy protection and model utility. The integration of multiple privacy techniques may introduce new attack vectors or vulnerabilities and require fine-tuning of various configuration parameters. Integrating multiple privacy techniques in an FL system requires additional development and maintenance efforts. The field of privacy enhancement technology in FL is still evolving, and standardization is limited. Addressing these weaknesses requires careful consideration, proper evaluation, and robust implementation of mixed, hybrid, and miscellaneous approaches. Balancing the complexity, performance impact, and security considerations is essential to leverage the strengths of these approaches while mitigating their weaknesses in the context of FL.

Thus, the trade-offs arise due to the combination of different techniques and the complexity of integrating them, including Complexity vs. Effectiveness, Privacy vs. Utility, Performance vs. Privacy, Compatibility and Interoperability vs. Customization, Security vs. Complexity, Standardization vs. Flexibility. Addressing these trade-offs requires careful evaluation, analysis of the specific use case, and understanding of the trade-offs within the context of the FL system. Regarding the adoption and integration of mixed, hybrid, and miscellaneous privacy enhancement approaches, the priorities, constraints, and goals to make informed decisions should be considered.

### 4.3. Research Question 3

The third research question is “What is the future of privacy enhancement methods in FL in healthcare systems?”

The third research question is answered based on the seven aspects as summarized in the literature reviewed in RQ1.

#### 4.3.1. Differential Privacy

Differential privacy provides an excellent solution for quantifying privacy guarantees of the learning algorithms for FL systems, it has been adopted by many leading technology companies, such as Apple, Google, and Facebook. The basic idea behind DP is the requirement of indistinguishable results by machine learning algorithms for any two datasets that differ by at most one individual’s data. As data from multiple participants are collected and shared, DP ensures the identity of any individual in the dataset cannot be reconstructed and hence provides privacy protection to user information.

The differential privacy approach provides an effective mechanism to safeguard the exchange of ML model parameters from clients to FL or edge server [27,28,29,31]. DP also poses all relevant characteristics to mitigate all risks associated with information leakage during model parameter exchange [26,30,32]. It exhibits all qualities that stop adversaries from reverse-engineering or inferring sensitive patient information from healthcare data by carefully controlling the amount of noise added during the FL model aggregation process.

As discussed before, the differential privacy technique provides data privacy by introducing noise that adversely affects the prediction accuracy of the ML algorithms used in FL systems. One way to achieve a balance of privacy and accuracy is by designing new aggregation algorithms that are optimized for differential privacy in FL. The basis existing aggregation techniques for FL, such as the Federated Averaging Algorithm (FedAvg), use randomized approaches to achieve differential privacy, but their performance may deteriorate in the presence of adversarial attacks. More sophisticated aggregation techniques need to be designed that can withstand the attacks while still providing strong privacy guarantees. FL systems generally involve a large number of participants, that are willing to collaborate without sharing their data. New aggregation algorithms and techniques must be scalable while having the capability to handle the computational challenges of differential privacy in FL. DP involves evaluations of privacy guarantees and the existing privacy evaluation metrics are not designed for distributed systems like FL. Hence, suitable and robust evaluation metrics are required that can account for the unique characteristics of the FL systems.

#### 4.3.2. Homomorphic Encryption

Homomorphic encryption provides privacy preservation for FL systems by allowing computations to be performed on encrypted data without compromising the privacy of the data. This approach is very effective for FL privacy preservation as most of the operations required by the FL systems like aggregation, model updates, and gradient calculations can be performed without accessing the raw data [34,35,36,37,38]. This encryption method provides a very relevant and suitable option for FL systems as even the cloud or edge servers performing the aggregation processes cannot access the data and only clients have access to the raw data. This decentralised control of the local data encourages clients to participate in FL systems without worrying about the patient information leakage in healthcare applications.

Homomorphic encryption can be computationally expensive, and performing computations on encrypted data requires significant computational resources. For FL applications where data is distributed and the computational capacity of the participants is not known, new algorithms are required that consider optimised use of computational resources at the participants. In this context, we need to consider the type of computations required by different ML algorithms and a suite of Homomorphic algorithms may be required to optimise computational efficiency for specific machine learning models, As discussed in the previous section, scalability and privacy-accuracy trade-off needs to be considered when FL specific Homomorphic algorithms are designed.

#### 4.3.3. Blockchain

In FL, blockchain technology has been adopted as a privacy-enhancing approach. In the decentralized environment, blockchain enables the secure and tamper-proof process for data sharing and computation using encrypted public keys. Therefore the participants’ data at the local level are better protected in terms of privacy and security. The existing work in this review presented the approaches including adding gradient [42], extra elements [43], hashed EHR address [44], and additional rules [45]. These approaches all require the second privacy-enhancing technique.

Four FL-chain healthcare applications [42,43,44,45] in this review prove that the blockchain technology is relevant to FL. The combined technologies effectively enhance privacy, security, transparency, collaboration, and governance. The biggest challenge is the computation overhead. To achieve maximum effectiveness, decisions need to be made regarding the design of smart contracts and consensus mechanisms, overall system design, acceptable training time, additional privacy enhancement techniques, regulations, governance, and so forth.

Although blockchain technology provides a high level of security, it is not inherently protected. There are still concerns that adversaries could link transactions to individual participants in the network. Future work also includes the investigation of alternative second privacy-preserving techniques, such as multi-party computation, or zero-knowledge proof, into an FL-chain system to achieve higher privacy and security.

Additionally, smart contracts, self-executing agreements stored on the blockchain, may automate certain aspects of the FL systems. Theoretically, those smart contracts facilitate the predefined rules, protocols, and incentives, ensuring compliance with data usage policies, distributing rewards if any, and managing the overall governance of the system. Additionally, smart contracts reduce the need for manual intervention and enhance efficiency. Smart contracts have only been mentioned in one of the existing works in this review [68]. It awaits further exploration in the future.

The evolving blockchain technology creates the concept of a token economy where the community’s revenue can be allocated to the users who create value in the system [69]. None of the four FL-chain applications in this review adopted a reward system. To achieve greater collaboration and participation, token-based economies or reward mechanisms can be implemented in blockchain-based FL healthcare systems.

#### 4.3.4. Hierarchical Approaches

In the category of hierarchical approaches in FL, existing work focused mainly on grouping clients in different levels or providing a hierarchical architecture to ensure even distribution of the network load among all connected devices and to minimise the effect of client movement or heterogeneity. These approaches are effective and relevant for enhancing privacy preservation in FL systems as they provide additional layers of control over data sharing among clients, edge and cloud servers. By leveraging hierarchical structures to organize clients and servers, privacy-preserving collaboration is facilitated [46,47,48].

For this approach, further work is required in order to secure sensitive participant information and minimise security threats from adversaries in the network. Secure communication mechanisms are vital to ensure that communication at different levels of the FL hierarchical system provides privacy of the data in an effective manner. Fine-grained privacy guarantees need to be incorporated into these approaches to provide fine-grained control over data sharing and participation. A dynamic hierarchical structure can build trust in the system as that allows clients to connect to servers that they know or trust. These approaches need a customised aggregation mechanism according to the applications’ requirements while providing privacy preservation. In addition, information flow management is crucial for ensuring privacy compliance and assurance against information leakage.

#### 4.3.5. Peer to Peer Sharing

P2PS is able to provide high privacy guarantees, improved data availability, distributed computing power, geographic distribution, collaborative learning, better scalability, and increased fault tolerance. There still has room to improve P2PS in FL. For example, considering the communication overhead and synchronization issues, developing new algorithms and protocols for communication and synchronization in FL is a promising area to reduce communication overhead and ensure efficient synchronization. Security and trust challenges, network and communication overhead, governance and control, and adoption and compatibility also need to be considered.

The implications of using P2PS technology in FL highlight the potential for improved privacy, enhanced collaboration, and distributed computing power. However, addressing security challenges, managing network overhead, establishing governance models, and ensuring compatibility are important considerations for successful implementation.

In addition, although the decentralized architecture for P2PS in FL is very important, the underlying architecture is a complex and dynamic process that needs to be optimized. Thus, to efficiently handle the computational requirements of machine learning tasks, it is critical to improve P2PS. Furthermore, developing privacy-preserving mechanisms is crucial to further improve privacy and security in P2PS. Besides, consensus mechanisms are a suitable solution for the P2PS network to reach agreement on data or models. New consensus mechanisms are needed to efficiently achieve agreement on large-scale datasets.

In conclusion, future work of P2PS should focus on developing new algorithms and protocols for communication and synchronization, optimizing the decentralized architecture, developing new privacy-preserving mechanisms, consensus mechanisms, and developing evaluation metrics. The effectiveness of P2PS technology in an FL system depends on the specific implementation, network characteristics, security measures, and governance mechanisms in place. Proper design and management of the P2P infrastructure, along with addressing potential challenges, contribute to maximising its effectiveness in FL scenarios.

#### 4.3.6. Intelligence on the Edge Device

As discussed in the Section 4.1, various techniques (such as the use of various ML and AI models, injection of noise, use of encryption techniques etc) have been used in the existing literature to improve the privacy of data in health systems. However, more work is needed in developing a new privacy protection scheme for FL especially considering the lightweight requirements for medical IoT devices used on the edge. Also, there is still room for improvement in privacy-preserving strategies, such as the incorporation of differential privacy, efficient encryption techniques, noise injection and secure multi-party computation for better results.

To summarise, we emphasise that intelligence on the edge device can play a big role in improving privacy and efficiency in FL. But there are significant challenges that need further attention, such as designing optimal infrastructure, and developing new privacy-preserving mechanisms and strategies. By addressing these challenges, we can develop more effective and efficient privacy-preserving techniques for FL on the Edge.

Besides privacy enhancement, intelligence on edge devices in FL systems holds significant relevance in various contexts and domains, including but not limited to scalability, real-time processing, bandwidth optimization, energy efficiency, and offline functionality. Intelligence on the edge devices in FL systems brings effectiveness through improved privacy protection, enhanced data security, scalability, reduced network latency, real-time decision-making, fault tolerance and resource efficiency. These benefits make intelligence on the edge a powerful approach, ensuring efficient and secure machine learning deployments in decentralized environments. By leveraging intelligence at the edge devices, FL systems could enable efficient and secure machine learning deployments.

#### 4.3.7. Mixed, Hybrid and Miscellaneous Approaches

The mixed, hybrid, and miscellaneous approaches have higher privacy guarantees compared to using a single technique alone by integrating multiple privacy techniques. Thus, it is important to combine different mechanisms to reduce the vulnerabilities and limitations of individual techniques, which is able to improve privacy preservation. These approaches can contribute to improved data confidentiality by encrypting data or performing computations without exposing the raw data, these approaches can reduce the risk of data leakage or unauthorized access. These approaches offer flexibility in tailoring privacy protection to specific requirements and constraints by customizing the privacy measures based on the sensitivity of the data, regulatory compliance needs, or the privacy preferences of participants.

However, the integration of multiple privacy techniques could increase computational complexity, interoperability, and standardization challenges. In addition, implementing and managing these hybrid approaches can be more complex than using a single technique. Evaluating the effectiveness and security of these approaches is challenging as they may require specialized evaluation methodologies and benchmarks to assess their impact on privacy, utility, and overall system performance.

These mixed approaches have shown high performance in FL by addressing the challenges associated with privacy preservation. They offer several relevances that contribute to the effectiveness and practicality of privacy protection in FL. First, they provide a comprehensive framework for privacy protection in FL by integrating multiple techniques to ensure that sensitive data remains secure and private throughout the FL process. They also offer flexibility and customization options to meet specific privacy requirements, enabling organizations to adapt the privacy measures to their unique use cases and compliance needs while achieving the desired level of privacy protection. They allow for optimizing the trade-off between privacy and utility in FL, ensuring that privacy enhancement does not compromise the effectiveness and utility of the FL system. The integration of multiple privacy techniques increases the resilience of the FL system against privacy attacks, helping protect against different types of privacy attacks, including inference attacks, membership inference attacks, or model inversion attacks. By employing a comprehensive privacy framework, organizations can demonstrate their commitment to protecting the privacy of individuals’ data. By combining different techniques, researchers can explore new ways to improve privacy protection and address emerging privacy challenges, driving the development of new privacy-enhancing mechanisms, algorithms, and protocols. In addition, researchers can combine their expertise and leverage the strengths of various privacy mechanisms, promoting collaboration across different disciplines, fostering the exchange of ideas, and accelerating progress in privacy-enhancing technologies.

The effectiveness of mixed, hybrid, and miscellaneous approaches to privacy enhancement technology in an FL system depends on several factors, including the specific techniques employed, the nature of the data, and the use case. While these approaches offer the potential for improved privacy protection, their effectiveness can vary. They should be evaluated on a case-by-case basis, taking into account the specific requirements, data characteristics, and threat landscape of the FL system. It is very important to have proper implementation, configuration, and validation to ensure the effectiveness of mixed, hybrid, and miscellaneous approaches to privacy enhancement in FL.

For future work, new decentralized FL models can be explored to reduce communication overhead and ensure efficient synchronization. Considering the model architecture is the key to combining different machine learning models, a proper optimization algorithm can be designed. In addition, privacy concerns still exist using the current hybrid FL, the use of encrypted data in FL is able to ensure the privacy and security of the data. This involves encrypting the data before sharing and performing computations on the encrypted data. It is necessary to develop a strategy by combining differential privacy, homomorphic encryption, and secure multi-party computation to further improve privacy and security. Furthermore, multiple privacy enhancement methods can be integrated into FL systems. Finally, as robust evaluation metrics can provide a standardized framework for measuring the effectiveness of P2P-sharing-based FL systems, it is vital to develop proper evaluation metrics in hybrid FL.

## 5. Conclusions

Machine learning, and in particular deep learning, has led to a wide range of innovations in the digital healthcare area. FL opens further opportunities in the novel research and business avenues by providing security and privacy for user data. This distributed machine learning technology enables participants, such as multiple hospitals, MIoT devices, or EHRs from various locations in healthcare systems, to perform training locally without sending data to the central server. This streamlines the healthcare process and improves healthcare decisions and diagnoses.

Given the lack of a comprehensive survey on the privacy enhancement methods in FL in healthcare systems, our review provides a detailed discussion and analysis in seven main areas: Differential Privacy, Homomorphic Encryption, Blockchain, Hierarchical Approaches, Peer to Peer Sharing, Intelligence on the Edge Device, and Mixed, Hybrid and Miscellaneous Approaches. Then, the possible future work for these seven areas to enhance privacy for FL in healthcare systems is identified and discussed.

The innovative FL applications in healthcare systems are still at a very early stage. It is crucial to assess the specific requirements, constraints, and trade-offs of utilizing additional privacy-enhancing techniques in an FL system. Exploring all approaches and building hybrid solutions that combine multiple techniques may help mitigate the limitations of each privacy-enhancing technique and optimize the overall performance of the system. Approaches like DP and HE provide significant privacy enhancements but add computational and governance overhead. On the other hand, more decentralized approaches like blockchain and P2PS provide robustness, scalability and fault tolerance with limited privacy guarantees. Therefore, while considering the strengths and limitations of all these approaches more research efforts are required for a one-fit-all method for all healthcare applications using the FL-based collaborative machine learning.

The P2PS can be used in FL systems where distributed computing power, scalability, privacy, fault tolerance, and geographical distribution are required. For intelligence on edge devices, they can be applied in FL systems where privacy, reduced communication, real-time processing, robustness, energy efficiency, and scalability are critical factors. While the mixed, hybrid, and miscellaneous approaches can be utilized in FL systems where complementary privacy techniques, fine-grained privacy control, balancing privacy and utility, flexibility and adaptability, robust defence against attacks, and compatibility and interoperability are needed.

In light of its highly personal nature, patient data is considered sensitive, confidential and private. The potential risks associated with its unauthorized access or disclosure. Although the inherent characteristics of FL architecture and additional privacy enhancement techniques, this intelligence builder faces ethical challenges. During the aggregation and transmission phases, sensitive patient data face the risks of data breaches, unauthorized access, identity theft, or even malicious modification. Sharing patient data across multiple institutions requires informed consent. Additionally, collaboration between multiple healthcare institutions requires accountability guidelines and governance structures to ensure ethical conduct throughout the FL process. Furthermore, biased data could affect FL training outcomes. Regular audits and validation processes in FL should be implemented to detect and address potential bias. Lastly, errors occurring in data transmission or training outcomes can erode the trust in the FL system. Therefore, it is crucial to run the FL-based healthcare systems in an ethical and trust environment.

We have a strong belief that technological advancements oriented from FL will enhance the healthcare systems globally, and the privacy enhancement methods presented in this review, as well as beyond this review, will propel FL to a new height.

## Figures and Tables

**Table 1:** Key topics and highlights of the existing review studies.

Paper	Key Topic	Highlights
Rieke et al. (2020) [1]	FL concept	Discussion on data heterogeneity, privacy and security, traceability and accountability, and system architecture in FL systems.
Xu et al. (2021) [7]	FL concept	Solution on the statistical challenges, system challenges, and privacy issues including secure multi-party computation and differential privacy in FL systems.
Shyu et al. (2021) [8]	FL concept	Evaluation on data partitioning characteristics, data distributions, data protection mechanisms, and benchmark datasets in FL systems.
Kumar and Singla (2021) [10]	FL in EHR	Comparison between different FL algorithms for different health sectors by using parameters such as accuracy, precision, recall and F-score.
Pfitzner et al. (2021) [9]	FL concept	Research into FL and its applicability for confidential healthcare datasets. Decentralized learning using a Blockchain or direct Peer-2-Peer network is excluded from the review.
Mothukuri et al. (2021) [15]	Security threats of FL	Comparison between privacy-specific threats and security threats in FL systems. Discussion on the security vulnerabilities and threats and privacy threats with their mitigation techniques.
Chowdhury et al. (2022) [13]	FL in Oncology	Discussion on FL applications and algorithms in the oncology space.
Nguyen et al. (2022) [16]	FL for EHR	Discussion on FL design from resource-aware FL, secure FL, privacy-aware FL to incentive FL and personalized FL.
Antunes et al. (2022) [12]	FL for EHR	Investigation on applications in the context of EHR, and further discussion on general architecture to FL with ML-enabled applications.
Ali et al. (2022) [14]	FL for IoMT	Focus on the IoMT network from the perspective of privacy. Discussion on architectures, from privacy-enabled FL, incentive-enabled FL for IoMT and FL-enabled digital twin for IoMT.
Our review	Privacy Enhancement for FL	Focus on the privacy enhancement methods used in FL in healthcare systems.

**Table 2:** Bibliography data source and parameters of the search.

Search Parameter	Target
Bibliography Data Source:	Scopus
Article Type:	Journal articles, conference papers, working papers, book section
Search On:	Title, Abstract, Keywords
Sorting on Returns:	Sort by Relevance
Publication Period:	Unlimited
Search Date:	28 August 2022

**Table 3:** Search keywords.

Component Index	Keywords
Component 1:	“federated learning”
Component 2:	“ehealth” OR “health”
Component 3:	“security” OR “privacy” OR “trust”

**Table 4:** Key strengths of privacy enhancement methods of the existing review studies.

Strength	DP	HE	BC	HA	P2PS	IE	HYA
Privacy	Yes	Yes	Yes	Yes	Yes	Yes	Yes
Security	Partial	Partial	Yes	Yes	Yes	Yes	Yes
Decentralization	Partial	Partial	Yes	Yes	Yes	Yes	Yes
Transparency	Yes	Yes	Yes	Yes		Yes	Yes
Data Governance		Yes	Yes	Yes	Yes	Yes	Yes
Collaboration	Yes	Yes	Yes	Yes	Yes	Yes	Yes
Scalability	Yes	Yes		Yes	Yes	Yes	Yes
Computational Capability	Yes	Yes		Yes	Yes	Yes	Yes
Data Transmission	Yes	Yes		Yes			Yes
Fault Tolerance		Yes	Yes		Yes	Yes	Yes
Energy Efficiency					Yes	Yes	
Improved Robustness						Yes	Yes
Smart Contract Automation			Yes				Maybe
Geographical Distribution			Yes		Yes	Yes	Yes

**Table 5:** Key weaknesses of privacy enhancement methods of the existing review studies.

Weakness	DP	HE	BC	HA	P2PS	IE	HYA
Scalability	Yes	Yes	Yes		Yes	Yes	Yes
Computational Overhead	Yes	Yes	Yes	Maybe		Yes	Yes
Transmission Overhead	Yes	Yes	Yes	Yes	Yes	Yes	Yes
Privacy Challenges	Partial	Partial	Yes	Partial	Yes	Yes	Yes
Design Complexity	Yes	Yes	Yes	Yes	Yes	Yes	Yes
Energy Consumption	Yes	Yes	Yes	Partial	Yes	No	Yes
Governance		Yes	Yes	Yes	Yes	Yes	Yes
Heterogeneous Devices					Yes	Yes	Yes
Data/Device Imbalance					Yes	Yes	Yes

## Data Availability

The data analyzed during this study are available via public bibliographic databases.

## Sample Availability

Samples of the compounds are available from the authors.

## Abbreviations

The following abbreviations are used in this manuscript:

MDPI	Multidisciplinary Digital Publishing Institute
DOAJ	Directory of open access journals
TLA	Three letter acronym
LD	Linear dichroism
